# Creating Porcine Biomedical Models Through Recombineering

**DOI:** 10.1002/cfg.404

**Published:** 2004-04

**Authors:** Margarita M. Rogatcheva, Laurie A. Rund, Kelly S. Swanson, Brandy M. Marron, Jonathan E. Beever, Christopher M. Counter, Lawrence B. Schook

**Affiliations:** 1 Department of Animal Sciences University of Illinois 329 Edward R. Madigan Laboratory 1201 W. Gregory Dr. Urbana IL 61801 USA; 2 Department of Veterinary Pathobiology University of Illinois Urbana IL 61801 USA; 3 Department of Pharmacology and Oncology Duke University Medical Center Durham NC USA

## Abstract

Recent advances in genomics provide genetic information from humans and other mammals (mouse, rat, dog and primates) traditionally used as models as well as new candidates (pigs and cattle). In addition, linked enabling technologies, such as transgenesis and animal cloning, provide innovative ways to design and perform experiments to dissect complex biological systems. Exploitation of genomic information overcomes the traditional need to choose naturally occurring models. Thus, investigators can utilize emerging genomic knowledge and tools to create relevant animal models. This approach is referred to as reverse genetics. In contrast to ‘forward genetics’, in which gene(s) responsible for a particular phenotype are identified by positional cloning (phenotype to genotype), the ‘reverse genetics’ approach determines the function of a gene and predicts the phenotype of a cell, tissue, or organism (genotype to phenotype). The convergence of classical and reverse genetics, along with genomics, provides a working definition of a ‘genetic model’ organism (3). The recent construction of phenotypic maps defining quantitative trait loci (QTL) in various domesticated species provides insights into how allelic variations contribute to phenotypic diversity. Targeted chromosomal regions are characterized by the construction of bacterial artificial chromosome (BAC) contigs to isolate and characterize genes contributing towards phenotypic variation. Recombineering provides a powerful methodology to harvest genetic information responsible for phenotype. Linking recombineering with gene-targeted homologous recombination, coupled with nuclear transfer (NT) technology can provide ‘clones’ of genetically modified animals.


**Comparative and Functional Genomics**
*Comp Funct Genom* 2004; **5**: 262–267.
Published online in Wiley InterScience (www.interscience.wiley.com). **DOI:** 10.1002/cfg.404

**Conference Review**

### Creating porcine biomedical models through recombineering

Margarita M. Rogatcheva[1], Laurie A. Rund[1], Kelly S. Swanson[1], Brandy M. Marron[1], Jonathan E. Beever[1], Christopher M. Counter[3] and Lawrence B. Schook[1,2]*

[1] *Department of Animal Sciences, University of Illinois, Urbana, IL 61801, USA*
[2] *Department of Veterinary Pathobiology, University of Illinois, Urbana, IL 61801, USA*
[3] *Department of Pharmacology and Oncology, Duke University Medical Center, Durham, NC, USA*

**Correspondence to:*
*Lawrence B. Schook, Department of Animal Science and Veterinary Biology, University of Illinois, 329 Edward R. Madigan Laboratory, 1201 W. Gregory Dr., Urbana, IL 61801, USA.*
*E-mail: schook@uiuc.edu*

Received: 16 February 2004
Accepted: 17 February 2004

### Abstract

**Recent advances in genomics provide genetic information from humans and other mammals (mouse, rat, dog and primates) traditionally used as models as well as new candidates (pigs and cattle). In addition, linked enabling technologies, such as transgenesis and animal cloning, provide innovative ways to design and perform experiments to dissect complex biological systems. Exploitation of genomic information overcomes the traditional need to choose naturally occurring models. Thus, investigators can utilize emerging genomic knowledge and tools to create relevant animal models. This approach is referred to as reverse genetics. In contrast to 'forward genetics', in which gene(s) responsible for a particular phenotype are identified by positional cloning (phenotype to genotype), the 'reverse genetics' approach determines the function of a gene and predicts the phenotype of a cell, tissue, or organism (genotype to phenotype). The convergence of classical and reverse genetics, along with genomics, provides a working definition of a 'genetic model' organism (3). The recent construction of phenotypic maps defining quantitative trait loci (QTL) in various domesticated species provides insights into how allelic variations contribute to phenotypic diversity. Targeted chromosomal regions are characterized by the construction of bacterial artificial chromosome (BAC) contigs to isolate and characterize genes contributing towards phenotypic variation. Recombineering provides a powerful methodology to harvest genetic information responsible for phenotype. Linking recombineering with gene-targeted homologous recombination, coupled with nuclear transfer (NT) technology can provide 'clones' of genetically modified animals. Copyright © 2004 John Wiley & Sons, Ltd.**

**Keywords:** **genomics; animal models; porcine; recombineering; BAC**

### Introduction

Comparative genomics provides an important criterion for creating animal models relevant to dissecting human diseases. Resolving complex human diseases is difficult (e.g. time course of disease onset in animals, expenses associated with human clinical experiments, ethical issues), and thus appropriate biomedical models must be developed and validated. Biomedical models are defined as 'surrogates for a human being, or a human biologic system, that can be used to understand normal and abnormal function from gene to phenotype and to provide a basis for preventive or therapeutic intervention in human diseases' [10,18]. In the past, researchers have used two main approaches to study human diseases. In one strategy, a human clinical disease is fully characterized and then the most appropriate animal model is chosen, based on criteria such as anatomical and/or physiological

Copyright © 2004 John Wiley & Sons, Ltd.

characteristics (biological relevance), cost and animal husbandry required. Another tactic has been to characterize naturally occurring or induced (by chemical or radiation exposure) mutant animals (most commonly the rat or mouse) and identify which human disease they resemble.

Integrating gene discovery and functional analysis is key to harvesting the 'genomic promise'. Validation of identified genes associated with QTL is essential. Because traditional validation approaches are time consuming, expensive and complicated by the diverse genetic backgrounds of non-inbred breeding stock, new methods of directly testing the causal effect of a given gene for a given phenotype are required. Because complex traits are polygenic (i.e. controlled by more than one genetic component), the ability to use a somatic cell target prior to NT to produce multiple gene substitutions into a single genetic background is essential for validating genetic interactions. Finally, by using somatic cell genomics, an *ex vivo* platform for expression profiling prior to NT, the number and associated costs of transgenic animals may be reduced.

Over the past decade, tremendous progress has been made with regard to the mapping and characterization of the swine genome. Moderate- to high-resolution genetic linkage maps containing highly polymorphic loci (Type II) have been produced using independent mapping populations [11,15]. Additionally, physical mapping methods, such as somatic cell hybrid analysis [12,19], *in situ* hybridization and ZOO-FISH [1] have been employed to enrich the Type I marker map and to perform comparative analysis with map-rich species such as the human and mouse. To date, over 5000 mapped loci are catalogued for the pig genome (**http://www.thearkdb.org**). Swine whole-genome radiation hybrid (WG-RH) maps also have been generated [6], resulting in yet another rapid increase in the number of loci mapped. Even more recently, the swine genomics community has acquired access to resources such as BAC libraries [5] to facilitate the production of high-resolution physical maps in specific chromosomal regions [13,14], and the construction of sequence-ready mapping resources for the porcine genome.

In current model systems (fruit fly, yeast, roundworm and mouse), functional genomics is supported by the ability to develop congenic inbred lines, cloning and creating mutants by either deletion or substitution of specific genes [17]. In pigs and cattle, there are neither inbred lines nor embryonic stem cell (ES) lines to create gain-of-function (GOF, knock-in) or loss-of-function (LOF, knockout) lines. In addition, the relatively long gestation time and costs of creating large breeding herds to map polygenic traits devoid of various background genes is not cost-effective with respect to validating QTL through breeding. Thus, there is an important need to develop *in vitro* correlates for transcription profiling (functional genomics and proteomics) similar to that developed for the worm, fly and mouse. This discovery platform must also allow capture of sequence information from comparative genomes (creating models to validate hypotheses with respect to gene–gene interactions associated with multigenic traits). It is also important that such an experimental system should allow the introgression of 'alleles' into germplasm, overcoming the current limitation of complicated targeting and deletion constructs and manipulation involved in constructing large pieces of DNA for homologous recombination (HR). Such a model for an integrated approach to build, test and refine cellular pathways resulting from specific perturbations (analysed by DNA microarrays) has recently been developed using *Saccharomyces cerevisiae* [17].

'Recombineering', or 'chromosomal engineering', permits directed genetic modification of genomic DNA and links gene discovery of a given phenotype with functional analysis (directly testing cause and effect) [2]. Recombineering provides a rapid method to genetically manipulate large DNA inserts cloned into BACs [16]. BACs have proved useful for cloning and maintenance of large DNA fragments in a *rec*$A^-$ genetic background that prevents genomic rearrangement. However, such host backgrounds also prevent the manipulation of insert DNA using conventional homologous recombination techniques [9]. Recently, a number of approaches involving modification of the host bacterium have been developed to permit BAC manipulation. These have included inducible promoters to permit transient expression of bacterial *rec*E and *rec*T genes or other analogous bacteriophage lambda ($\lambda$) genes (*exo* and *beta*) [20]. Recently an *Escherichia coli* strain harbouring a defective $\lambda$-prophage has been developed that promotes high BAC recombination frequencies.

Copyright © 2004 John Wiley & Sons, Ltd. 

In order to make this system suitable for BAC manipulation, the *E. coli* strain DY380 was generated by introducing the λ prophage into the BAC host strain DH10B [16]. The λ prophage provides a rapid single-step method to generate subtle changes in any gene in BAC clones using oligonucleotides as targeting vectors. By using a PCR-based selective amplification screen to identify targeted clones, this system enables the generation of single-base changes, deletions (up to 1.93 kb), and the insertion of unique sequences in different regions of a BAC containing *Brca2*. This system has since been used to generate BAC transgenic mice using a *Brca2*-deficient genetic background [7]. In addition, the ability to insert the fusion tag FLAG™, that consists of eight amino acids, including an enterokinase-cleavage site [4], into the *Brca2* gene provided a unique method to monitor gene expression.

In order for this experimental approach to be successful, DNA sequence information of relevant genomic regions containing genes of interest must be accessible. In our laboratory, we have targeted genomic sequencing of chromosomal regions that either contribute to resolving complex phenotypes (QTL) or act as biomedical research models. Regions of interest include the porcine MHC class I, myostatin, neurofibromatosis (NF-1) and the ataxia-telangiectasia (AT) genes. In this review, we describe how we have utilized the porcine BAC 403O5 (from the RPCI-44 library) that contains the myostatin gene to demonstrate our ability to introduce small and large insertions and to monitor the expression of recombineered genes in transfected fibroblasts.

### Genetic modifications of BACs

Using the recombinogenic *E. coli* strain DY380, we have rapidly generated multiple genetic modifications in BAC clones, harbouring the porcine myostatin gene [13]. PCR-derived ssDNA fragments were used to delete a 68 bp fragment from exon 3 of the porcine myostatin gene. A deletion in this region disrupts gene expression and blocks synthesis of the myostatin protein, resulting in a double-muscling phenotype. In order to create a targeted deletion, a 140 bp ssDNA targeting vector was constructed using a 100 bp synthetic template (50 bp flanking the deletion site in both directions) and 40 bp oligo 5′ and 3′ primers each of 20 bp flanking the 100 bp template and 20 bp overlapping the template. In the first set of experiments we used PCR screening with primers, designed using BAC sequencing information, flanking the deletion site of the gene. Upon analysis following recombineering, we identified PCR pools having both wild-type and recombineered product and demonstrated a recombineering deletion efficiency of 1 recombinant/470 electroporated cells. This is a lower targeting efficiency than was obtained in a similar experiment by Swaminathan *et al.* [16], where they introduced a deletion into exon 11 of the murine *Brca*2 gene with a 1/120 targeting frequency. Thus, we re-screened our pools using the mismatch amplification mutation assay (MAMA-PCR) and the annealing temperature was adjusted so that a PCR product would only be amplified from BACs having a deletion, whereas a wild-type BAC would not be amplified. This approach permitted the detection of 1 recombinant/170 electroporated cells. DNA sequencing of the recombinant clones confirmed that the targeted deletion occurred in the desired location.

### Recombineering inserts into porcine BACs

The ability to generate small insertions is a key step in using the recombineering methodology to develop an animal model. Experiments were therefore designed to determine the targeting precision of inserting specific short sequences into a particular BAC region. The 24 bp sequence encoding the FLAG™ octapeptide, which when attached to the C-terminus of a protein can be used as a tag to distinguish between normal and modified protein and monitor protein movement within a cell as well as for protein purification by affinity chromatography, was utilized [13]. An oligonucleotide targeting vector (164 bp) to attach the FLAG™ tag at the C-terminus of myostatin was synthesized by PCR using two synthetic 94 bp oligonucleotides, which served as template and primer simultaneously. The targeting vector thus had 70 bp arms homologous to target regions flanking both the 5′ and 3′ end of the insertion site in the BAC encoding myostatin, and the 24 bp FLAG™ sequence. The recombinants were identified in cultured pools and then as individual colonies by FLAG™-specific PCR, with a 1/260 targeting efficiency. DNA sequencing of three PCR-positive individual clones showed that the targeting was specific and that no mutations

Copyright © 2004 John Wiley & Sons, Ltd.

were induced in sequences flanking the insertion site (500 bp in both directions).

### Recombineered target expression in somatic cells

Another vital component in developing a recombineering platform is the ability to use the recombineered BACs to introduce targeted changes in fibroblast and other somatic cells. These studies were designed to demonstrate (a) that larger, complex gene constructs could be developed and (b) that transient gene expression could be monitored following BAC transfection into somatic cells. This approach requires either a selection marker such as an antibiotic resistance gene or another selective marker such as green fluorescent

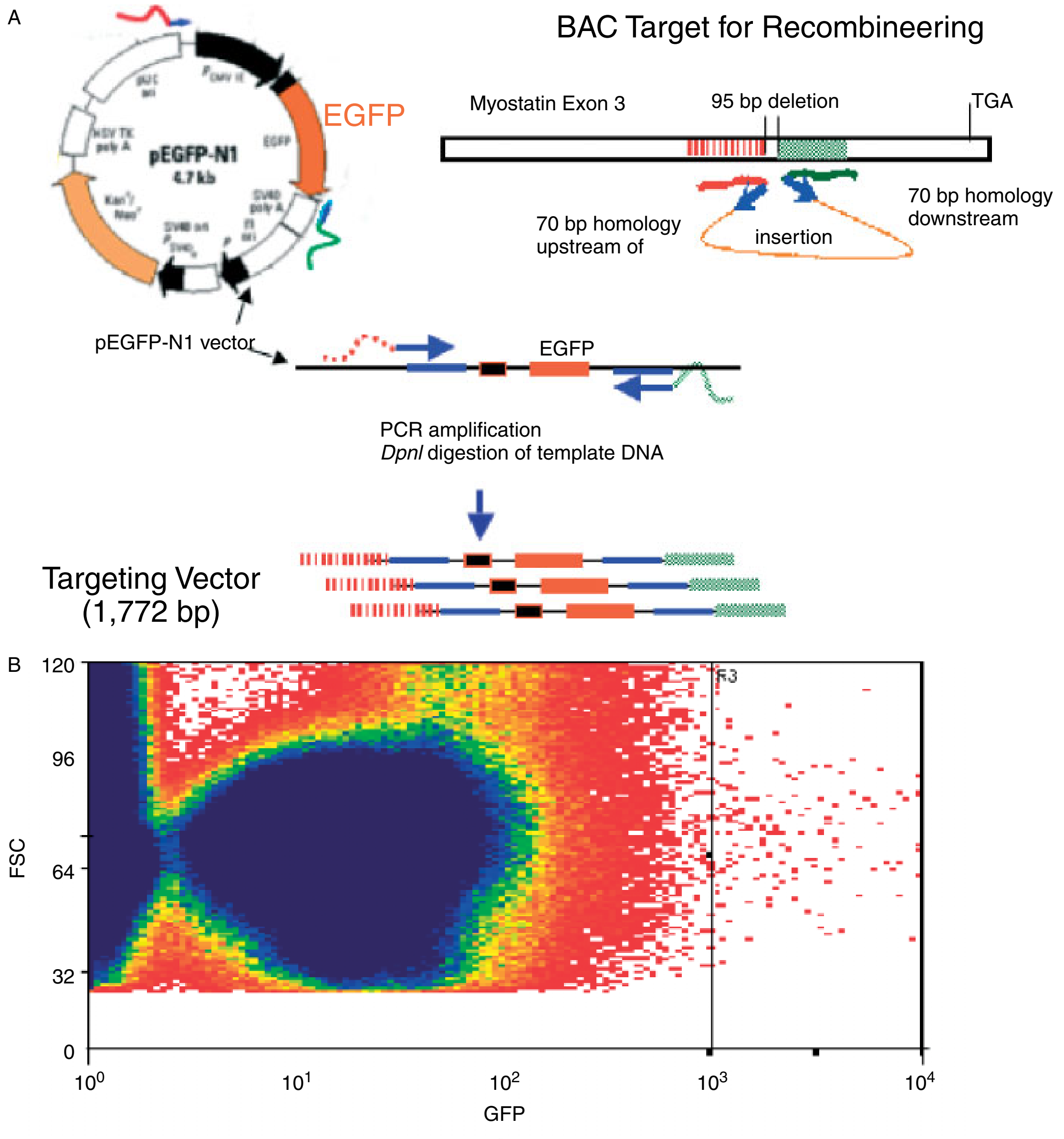

| Region | Count | % | Mean |
|---|---|---|---|
| Total | 1,173,125 | 100 | 15 |
| R3 | 162 | 0.01 | 2,626 |

Copyright © 2004 John Wiley & Sons, Ltd. 

**Figure 1.** (a) Constructing a targeting vector for EGFP insertion. The targeting vector was constructed using 70 bp arms homologous to sequences upstream and downstream of the insertion site (within exon 3 of the porcine myostatin gene). The pEGFP-N1 vector (Clontech) was used to amplify the targeting cassette containing the human cytomegalovirus immediate early promoter. The resulting recombineered vector was PCR-amplified and then treated with *DpnI*. The *DpnI* restriction enzyme cleaves the methylated GATC DNA of the pEGFP-N1 vector, thus preventing contamination with the EGFP expression vector in the recombineered preparation. As illustrated, the targeting vector used for recombineering is approximately 1.7 kb. (b) EGFP gene expression monitored by flow analysis of transfected porcine fetal fibroblasts. The targeting vector was used to insert EGFP into the myostatin containing BAC (175 kb). *E. coli* DY380 clones containing the modified myostatin–EGFP (recombineered BACs) were grown overnight and BAC DNA was extracted using a Nucleobond AX kit. Porcine fetal fibroblasts (8HY17F) were then electroporated with BAC DNA (10 μg). Electroporations using different conditions for EGFP expression were used to establish the amount of BAC and the optimal time of expression. Thus, cells were grown for 3 days and then sorted by flow cytometry to estimate the percentage of cells transfected and the level of transient gene expression protein. We have chosen the enhanced green fluorescent protein (EGFP) as a selection marker (Figure 1a). During the screening of transfected mammalian cells, EGFP expression can be used in conjunction with flow cytometric cell sorting to select for homologous recombinants. Using a vector containing the EGFP and its promoter and without a polyA tail, we constructed a 1592 bp sequence coding for EGFP. A 1772 bp targeting vector was amplified from the plasmid pEGFP-N1 (Clontech) using 90 bp PCR primers. Specific PCR amplification using pooled cells was able to detect recombinants in 4/190 pools, yielding a targeting efficiency for the large targeting cassette (EGFP) of 1/775. Although a higher targeting efficiency is observed for small oligonucleotide cassettes (deletion and FLAG™), it is still promising that such a large insert could be incorporated in a single step without the requirement of additional antibiotic resistance or other selection markers.

The recombineered BAC (EGFP–myostatin fusion gene) was then used to transfect fetal porcine fibroblasts to demonstrate the ability of recombineered genes to be expressed. As shown in Figure 1b, we were able to demonstrate the expression of EGFP by fetal fibroblasts as measured by flow cytometry. This study showed the efficiency of transient expression of recombineered myostatin BAC (approximately 170 kb) to be around 0.01%, which is consistent with previous studies [8]. These results suggest that insertions longer than 1000 bp can be successfully recombineered into a gene of interest. Constructs generated by this approach could also be used to develop specifically modified somatic cells for conditional expression, GOF or LOF studies.

### Discussion

This review summarizes our development of a somatic cell technology platform that integrates the recombineering of genomic DNA with the subsequent targeted gene manipulation of somatic cell lines. This platform provides a novel approach for dissecting physiological pathways using defined genetic systems *in vitro* while avoiding the cost of validation in non-inbred animals and 'genetic noise' from various background genes. The use of gene-targeted fibroblasts provides a suitable nuclear donor for use in nuclear transfer to generate unique swine germplasm for generating biomedical models. The targeting efficiencies observed in this study for FLAG™ insertion and deletions are comparable to those observed using a mouse model [7,16]. Our attention is now focused on developing site-specific recombination systems to permit targeted allelic substitution in porcine BACs. This will permit us to target gene function in somatic fibroblast lines and evaluate downstream events prior to *in vivo* studies. As previously reported by Montigny *et al.* [8], we were able to demonstrate that BAC DNA purity and concentration did affect transfection efficiency. Furthermore, although the BAC construct used here was extremely large (>175 kb), we were able to demonstrate transfection and expression profiles consistent with other studies [8].

### Acknowledgements

This work was partially supported by grants from the USDA–National Research Initiative (2002-35205-12712), the USDA Cooperative State Research Service (AG2002-34480-11828) and the USDA Agricultural Research Service (Agreement No. 58-5438-2-313). The authors wish to recognize the contributions and assistance of E. Forsberg,

Copyright © 2004 John Wiley & Sons, Ltd. 

Infigen. The authors also acknowledge the support and assistance of N. Copeland and N. Jenkins (NCI).

### References

1. Chowdhary BP, Raudsepp T, Fronicke L, Scherthan H. 1998. Emerging patterns of comparative genome organization in some mammalian species as revealed by Zoo-FISH. *Genome Res* **8**: 577–589.
2. Copeland NG, Jenkins NA, Court DL. 2001. Recombineering: a powerful new tool for mouse functional genomics. *Nature Rev* **2**: 769–779.
3. Dow JAT, Davies SA. 2003. Integrative physiology and functional genomics of epithelial function in a genetic model organism. *Physiol Rev* **83**: 687–729.
4. Einhauer A, Jungbauer A. 2001. The FLAG™ peptide, a versatile fusion tag for the purification of recombinant proteins. *J Biochem Biophys Meth* **49**: 455–465.
5. Fahrenkrug SC, Rohrer GA, Freking BA, *et al.* 2001. A porcine BAC library with tenfold genome coverage: a resource for physical and genetic map integration. *Mamm Genome* **12**: 472–474.
6. Hawken RJ, Murtaugh J, Flickinger GH, *et al.* 1999. A first generation porcine whole-genome radiation hybrid map. *Mamm Genome* **10**: 824–830.
7. Lee EC, Yu D, Martinez de Velasco J, *et al.* 2001. A highly efficient *Escherichia coli*-based chromosome engineering system adapted for recombinogenic targeting and subcloning of BAC DNA. *Genomics* **73**: 56–65.
8. Montigny WJ, Phelps SF, Illenye S, Heintz NH. 2003. Parameters influencing high-efficiency transfection of bacterial artificial chromosomes into cultured mammalian cells. *BioTechniques* **35**: 796–807.
9. Muyrers JPP, Zhang Y, Benes V, *et al.* 2000. Point mutation of bacterial artificial chromosomes by ET recombination. *EMBO Rep* **1**: 239–243.
10. NRC (National Research Council). 1998. *Biomedical Models and Resources: Current Needs and Future Opportunities*. National Academy Press: Washington, DC.
11. Paszek AA, Wilkie PJ, Flickinger GH, *et al.* 1999. Interval mapping of growth in divergent swine cross. *Mamm Genome* **10**: 117–122.
12. Rettenberger G, Klett C, Zechner U, *et al.* 1995. Visualization of the conservation of synteny between humans and pigs by heterologous chromosomal painting. *Genomics* **26**: 372–378.
13. Rogatcheva MB, Rund LA, Beever JE, Counter CM, Schook LB. 2002. Recombineering pig BACs for somatic cell genomics. *Int Soc Animal Genet* A022.
14. Rogel-Gaillard C, Bourgeaux N, Billault A, Vaiman M, Chardon P. 1999. Construction of a swine BAC library: application to the characterization and mapping of porcine type C endoviral elements. *Cytogenet Cell Genet* **85**: 205–211.
15. Rohrer GA, Alexander LJ, Hu Z, *et al.* 1996. A comprehensive map of the porcine genome. *Genome Res* **6**: 371–391.
16. Swaminathan S, Ellis HM, Waters LS, *et al.* 2001. Rapid engineering of bacterial artificial chromosomes using oligonucleotides. *Genesis* **29**: 14–21.
17. Trey R, Vesteinn T, Ranish JA, *et al.* 2001. Integrated genomic and proteomic analyses of a systemically perturbed metabolic network. *Science* **292**: 929–934.
18. Tumbleson M, Schook LB. 1996. *Advances in Swine in Biomedical Research*. Kluwer Academic: New York.
19. Yerle M, Echard G, Robic A, *et al.* 1996. A somatic cell hybrid panel for pig regional gene mapping characterized by molecular cytogenetics. *Cytogenet Cell Genet* **73**: 194–202.
20. Zhang Y, Buchholz F, Muyrers JPP, Stewart AF. 1998. A new logic for DNA engineering using recombination in *Escherichia coli*. *Nature Genet* **20**: 123–128.

Copyright © 2004 John Wiley & Sons, Ltd. 
